# Whey protein supplementation reduced the liver damage scores of rats fed with a high fat-high fructose diet

**DOI:** 10.1371/journal.pone.0301012

**Published:** 2024-04-04

**Authors:** Aslı Yiğit Ziolkowski, Nurgül Şenol, Rahime Aslankoç, Gülhan Samur

**Affiliations:** 1 Faculty of Health Sciences, Nutrition and Dietetics Department, Süleyman Demirel University, Isparta, Turkey; 2 Faculty of Medicine, Department of Physiology, Süleyman Demirel University, Isparta, Turkey; 3 Faculty of Health Sciences, Nutrition and Dietetics Department, Hacettepe University, Ankara, Turkey; Jordan University of Science and Technology Faculty of Medicine, JORDAN

## Abstract

Different functional foods with bioactive nutrients are being explored for the management of NAFLD. Whey proteins are rich in bioactive peptides and are suggested to show antioxidant and anti-inflammatory effects. We aim to test the hypothesis that the whey protein supplementation following a high fat-high fructose (HFHF) diet would protect against liver damage, inflammation, endotoxemia and steatosis in male Wistar rats. 36 rats were randomized into four groups for 8 weeks as the HFHF diet group, HFHF diet and whey protein isolate (WPI-200mg/kg/day) group (HFHF+WPI), control (C) group, and C+WPI (200mg/kg/day) group. Rats fed with a HFHF diet had higher final body weight compared to C and C+WPI groups (p = 0.002). Thus, WPI showed no significant effects for the body weight of rats with a HFHF diet. On the other hand, the HFHF+WPI group had significantly lower abdominal circumference when compared with the HFHF group (p<0,001). Higher serum CRP levels were observed in the groups with a HFHF diet (p<0,001) and WPI supplementation showed no effects on CRP levels. Whey protein supplementation resulted with lower total liver damage score in HFHF+WPI group compared with the HFHF diet group (p<0,001). Conversely, higher liver damage scores were observed with the C+WPI group compared to C group (p<0,001). HFHF diet resulted with higher expression of TLR-4 in the liver meanwhile WPI supplementation showed no effects on liver TLR-4 expression. We observed higher colon Occludin expression in HFHF+WPI and C+WPI groups compared with HFHF and C groups (p<0,001). Our results showed that, whey protein supplementation might help improve liver damage associated with a high fat-high fructose diet and increase the expression of Occludin in the small intestine and colon.

## 1. Introduction

Non-alcoholic fatty liver disease (NAFLD) is an important public health problem affecting 25% of the global population [1]. NAFLD is characterized by a wide spectrum of disorders ranging from simple steatosis to ongoing inflammation and fibrosis that is associated with non-alcoholic steatohepatitis (NASH) [2,3]. NAFLD is diagnosed by the presence of steatosis and abnormal fat deposition in the absence of a history of alcohol consumption or other significant liver disease [4]. NAFLD is recognized as the leading cause of chronic liver disease and NASH is seen as a major risk factor for liver cirrhosis and hepatocellular carcinoma [5–7]. Metabolic, genetic, epigenetic, immune and environmental mechanisms interact in the pathogenesis of NAFLD. In recent years, changes in the intestinal microbiota have been associated with the development of NAFLD [8–10]. The link between the intestinal microbiota and liver, known as the gut-liver axis, is suggested to play an important role in NAFLD and NASH [11,12]. The intestinal epithelium acts as a natural barrier that selects for beneficial substances and ensures the removal of potentially harmful elements. Tight junctions between epithelial cells are special intercellular structures that assist in this control. Disruption of tight junctions can result in increased intestinal permeability [13]. This disruption increases the passage of bacterial products, especially LPS (lipopolysaccharide), which is a component of the cell membrane of gram-negative bacteria, into the blood [11]. In this case, hepatic TLR-4 (toll-like receptor-4) is activated and inflammatory processes are initiated with the production of pro-inflammatory cytokines such as TNF-α (tumor necroes factor-α), IL-1β (interleukin 1β), and IL-6 (interleukin-6). This inflammatory response induces the production of profibrotic factors by hepatic stellate cells. It also disrupts mitochondrial β-oxidation and causes an increase in hepatic steatosis and hepatic inflammation [14]. As a result, endotoxemia and low-grade chronic inflammation develops and exacerbates the course of hepatic steatosis and cellular damage observed in NAFLD [15,16].

Once seen as a dairy byproduct from cheese production, whey proteins are now recognized as a high-quality nutrient source [6]. Whey proteins are rich in branched-chain amino acids and sulfur-containing amino acids [17]. A broad spectrum of functionality has been attributed to whey proteins, including antioxidant and anti-inflammatory effects, appetite suppression, blood-pressure-lowering effects, and immunomodulation. It is suggested that whey proteins provide antioxidant and anti-inflammatory effects due to their bioactive peptide content. In this respect, whey proteins could be used as a potential functional food for the dietary management of non-communicable diseases [6,18]. Recent studies have demonstrated the potentially beneficial effects of whey proteins on obesity by increasing insulin sensitivity and satiety [19–21]. Similarly, whey proteins might show potential positive effects on liver steatosis with its antioxidant effects, anti-inflammatory effects and effects on insulin sensitivity and satiety effects [6]. A study conducted on C57Bl/6J mice revealed that whey protein rich in lactoperoxidase, lactoferrin, growth factors and immunoglobulins, dose dependently prevented weight gain and protected against the fatty liver formation during ad libitum feeding after weight loss [22].

In this study, we aim to test the hypothesis that the whey protein supplementation simultaneously with a high fat-high fructose (HFHF) diet would protect against liver damage, inflammation, endotoxemia and steatosis. We also aimed to explore the effects of whey protein supplementation on the gut-liver axis, by examining the expression of TLR-4 in liver and Occludin in colon and small intestine.To investigate the potential effects of whey proteins, we conducted an experimental model of liver steatosis, feeding Wistar rats with a high-fat and high fructose (HFHF) diet while simultaneously administrating whey protein with oral gavage.

## 2. Methods and materials

### 2.1. Ethical statement

The care of the experimental animals and the experimental protocol were performed at Süleyman Demirel University Experimental Animal Production and Experimental Research Laboratory (HÜDAL) between September 21, and November 17, 2022. The euthanasia and collection of blood and tissue samples from the experimental animals were also performed at HÜDAL. This project proposal was was approved by the Animal Experiments Local Ethics Committee of Süleyman Demirel University (HADYEK-dated 06/01/2022, numbered 01/04). All procedures involving animals were conducted in accordance with the Guide for the Care and Use of Laboratory Animals.

### 2.2. Animals and experimental procedure

The experimental animals used in the study were 12-week-old male Wistar rats (200–250 gr weight) produced in HÜDAL. Animals were kept in HÜDAL at an ambient temperature of 21±2°C and a humidity of 55–60%, with a 12-hour dark and 12-hour light cycle. After an adaptation period of one week, 36 rats were randomized into 4 experimental group with nine rats per group. The four groups were: (1) HFHF diet with gavage of whey protein isolate (WPI); (2) HFHF diet with gavage of distilled water; (3) control diet with gavage of WPI; and (4) control diet with gavage of distilled water. A high-fat diet (45% kcal of fat) was provided from a company (ARDEN research, Ankara) that offered food in pellets and the drinking water was prepared with 10% of fructose syrup (F55). The control diet was also provided from ARDEN research. The composition of the diets is given in the **Table 1**. The dose of whey protein isolate was 200 mg/kg/day as previously reported to be sufficient to have positive effects [23,24]. The composition of the whey protein isolate is given in the **S1 Table**. Prior to administration, an appropriate amount of WPI was dissolved in distilled water. WPI solution or pure distilled water (0,5 ml) was administered with an oral gavage daily at the same time to all groups. All animals had access to food and water ad libitum. The weight of the rats was measured before the start of the intervention and weekly until the end of the experimental procedure. After 8 weeks of the intervention period, animals were euthanized after 12 hours of fasting. Anesthesia was administered by intraperitoneal injection of a ketamine-xylazine combination of 90 mg/kg-10 mg/kg, respectively. Blood samples were taken by cardiac exsanguination method and the necessary organs were removed after euthanasia. The liver, small intestine, and colon were removed and necessary sections were prepared for histopathological examination.

**Table 1:** Composition of experimental diets.

	Control Diet	High Fat diet*
	g	g
Casein, 90 Mesh	233,46	233,20
L -Cystine DL methionine	4,29	3,49
Corn Starch	333	84,88
Maltodextrin	116	116,60
Sucrose	5,168	201,48
Cellulose, BW200	60	58,30
Soybean Oil	58	29,15
Butter	0	206,96
Mineral Mix S10026	11,66	11,66
DiCalcium Phosphate	15,15	15,15
Calcium Carbonate	6,41	6,41
Potassium Citrate, 1 H2O	19,23	19,23
Vitamin Mix V10001	11,66	11,66
Choline Bitartrate	2,33	2,33
Dye	0,05	0,05
Protein	24	24
Carbohydrate	45	41
Fat	5,5	24
	kcal%	kcal%
Protein	30	20
Carbohydrate	55	35
Fat	15	45
Energy	3,2 kcal (1 g)	4,73 kcal (1 g)

### 2.3. Anthropometrical measurements

On the day of euthanasia, the body weights of the animals were measured. The naso-anal length and abdominal circumference of the rats were measured under anesthesia. The abdominal circumference (AC), and naso-anal length measurements was taken as previously described [25]. The Lee index was calculated for the assessment of obesity by dividing the cube root of the weight by the naso-anal length as previously described. Body mass index (BMI) was calculated by dividing body weight (g) by the square of the height (cm^2^) [25].

### 2.4. Biochemical assessments

Blood samples were transferred to 8.5 ml serum separator tubes, then centrifuged at 4000 rpm for 10 minutes. The plasma samples were transferred to 1.5 ml Eppendorf tubes and stored at -80°C until the day of analysis. For the homogenization of the liver tissue, firstly the samples were placed in phosphate buffer solution. Tissue samples were then fragmented with a homogenizer (IKA Ultra-Turrax T25 Basic, Labortechnik) and a sonicator (Bandelin HD2070). The obtained homogenate was centrifuged at 10.000 rpm for 10 minutes at 4°C and then stored at -80°C until the day of analysis. The biochemical markers and serum levels of LPS was analyzed with rat ELISA (Enzyme-linked Immunosorbent Assay) kits (BT LAB, China), according to the manufacturer’s instructions. We examined the LPS, IL-6, and TNF-a levels in serum and liver samples. Analysis of total cholesterol, triglycerides, alanine transaminase (ALT), aspartate transaminase (AST) and C-reactive protein (CRP) was performed on serum samples.

### 2.5. Histopathology

Histological examinations were performed on tissue samples taken from the liver, small intestine, and colon. Tissue samples were fixed in 10% formaldehyde then embedded in paraffin. Sections of 5 μm thickness were cut from the paraffin-embedded tissues. Sections were stained with hematoxylin and eosin (H&E) for general histological examinations. Images were taken with a light microscope (Leica DM 500, Germany). The histopathological examination of the liver tissue was performed according to Knodell scoring. Scores were given on the following basis: 0 = no pathological damage, 1 = little or no damage, 2 = moderate damage, 3 = significant damage, 4 = very severe damage [26]. Total liver damage score was calculated as the sum of the following scores: hydropic degeneration, mononuclear cell infiltration, sinusoidal dilatation, necrotic areas, congestion, and edema. The severity of steatosis was graded as 0–3 according to the Brunt classification (0 = <5% steatosis, 1 = 5–33% steatosis, 2 = 33–66% steatosis, 3 = >66% steatosis) [27]. The histological damage score of the small intestine tissue was evaluated according to the criteria set by Howarth et al. [28].

### 2.6. Immunohistochemical analysis

The expression of TLR-4 in liver, and Occludin in intestine and colon were determined by streptavidin peroxidase immunohistochemical staining. Deparaffinized tissues were washed with PBS (phosphate buffered saline) and treated with hydrogen peroxide solution for 5 minutes. Tissues were placed in 1 M sodium citrate solution (pH 6.0) for 12 minutes in a microwave oven to reveal antigenic receptors. Tissue sections were washed several times with PBS and treated with Ultra V Block solution for 5 minutes to prevent non-specific antibody binding, followed by incubation with primary antibodies (TLR-4 and Occludin) for 60 minutes. The tissues were then washed with PBS and incubated with secondary antibody (biotinylated Goat Anti-Polyvalent) for 30 minutes. Afterwards, the tissues were incubated with streptavidin peroxidase for 30 minutes. Samples were treated with the DAB Substrate Kit solution and were observed (with X 40 magnification) with a light microscope (Leica DM 500, Wetzler, Germany). The severity and extent of cytoplasmic immunostaining were scored semi-quantitatively on a scale of 0–3. (0 = none, 1 = slight, 2 = moderate, 3 = severe).

### 2.7. Statistical analyses

IBM SPSS version 23 was used for the evaluation of the statistical data. Graphical approaches and normality tests (Shapiro-Wilk test) were used for the conformity of the data to the normal distribution. Statistical significance was evaluated as p<0.05 level in all analyzes. One-way analysis of variance (One-way ANOVA) test was performed in independent groups, provided that they were compatible with normal distribution. Tukey HSD or Tamhane post hoc tests were used according to the variance distribution in pairwise comparisons. In case of non-conformity with normal distribution, Kruskal-Wallis analysis was performed. Repeated measures of variance analysis (repeated measures ANOVA) were used to compare the body weight of the groups during the experiment.

## 3. Results

A total of 36 rats (nine rats per group), were included in the study. The groups were: HFHF+WPI, C+WPI, HFHF, and C. The experimental protocol lasted 8 weeks.

### 3.1. Effects of whey protein isolate on anthropometrical indices

Initial and final weight, total weight gain, naso–anal length, Lee index, BMI, abdominal circumference measurement, and the weekly and daily weight gains of rats are given in **Table 2**. At the beginning of the experiment, there was no difference in body weight between the groups (p>0.05). Final body weight of the HFHF group were significantly higher compared with the C+WPI and C groups. The HFHF+WPI group had significantly higher body weight compared to the C+WPI group. The HFHF group had a higher Lee index compared with the C group (p<0.05). HFHF and HFHF+WPI groups had higher BMI compared with the C group (p<0.05). The abdominal circumference of the HFHF group was higher compared with the other groups (p<0.05). HFHF and HFHF+WPI groups had higher values for both weekly and daily weight gain compared with C and C+WPI groups (p<0.05).

**Table 2:** Anthropometric indices of male rats exposed to C, C+WPI, HFHF, or HFHF+WPI diets.

	C	C+WPI	HFHF	HFHF+WPI	p
Initial weight (g)	246,33 ± 13,08	250,11 ± 4,53	245,33 ± 5,46	248,33 ± 6,52	,976
Final weight (g)	377,00 ± 15,93 ^bc^	367,33 ± 7,34^b^	433,66 ± 15,62^a^	425,00 ±12,61 ^ac^	**,002**
Total weight gain (g)	130,66 ± 8,46^b^	117,22 ± 4,83^b^	188,33 ± 12,55^a^	176,66 ± 8,89^a^	**,000**
Naso-anal length (cm)	25,66 ± 0,33	24,88 ± 0,26	25,88 ± 0,35	25,88 ± 0,26	,083
Abdominal circumference (cm)	16,77 ± 0,14^a^	16,77 ± 0,22^a^	18,77 ± 0,27^b^	17,66 ± 0,33^a^	**,000**
LEE index	281,07 ±2,45^a^	287,82 ± 2,77^ab^	292,16 ± 2,23^b^	290,32 ± 3,15^ab^	**,033**
BMI (g/cm^2^)	0,57 ± 0,01^b^	0,59 ± 0,01^ab^	0,64 ± 0,01^a^	0,63 ± 0,01^a^	**,003**
Weekly weight gain (g)	16,33 ± 1,05^b^	14,65 ±0,60^b^	23,54 ± 1,56^a^	22,08 ± 1,11^a^	**,000**
Daily weigh gain (g)	2,33 ± 0,15^b^	2,09 ± 0,08^b^	3,36 ± 0,22^a^	3,15 ± 0,16^a^	**,000**

**C**, Control diet; **HFHF**, high fat-high fructose diet; **HFHF +WPI**, high fat-high fructose diet + whey protein isolate; **C+WPI**, Control diet+ whey protein isolate. Results were determined by one-way analysis of variance (One-Way ANOVA) and expressed as mean with standard error of mean (SEM). Tukey HSD test was used as post-hoc test in pairwise comparisons. Different letters indicate statistical significance.

**Fig 1:**
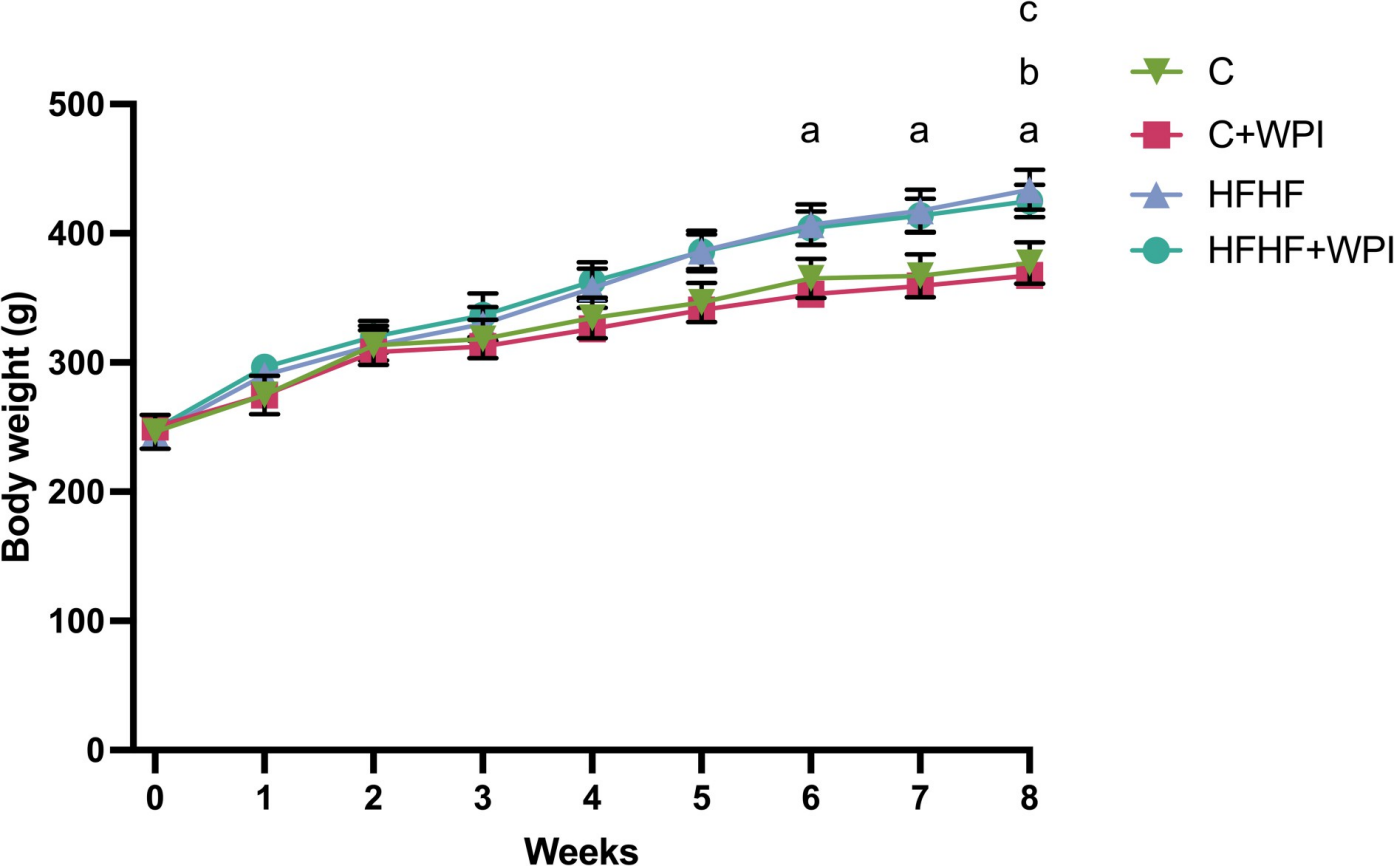
Whey protein supplementation had no effects on the body weight changes between the male rats fed C, C+WPI, HFHF, or HFHF+WPI diets. The mean body weight of the HFHF group was significantly higher than the C+WPI group at 6^th^, 7^th^ and 8^th^ weeks (p<0.05). The mean body weight of the HFHF+WPI group was significantly higher than the C+WPI group in the 8^th^ week while the body weight of the HFHF group was significantly higher both the C and C+WPI groups (p<0.05). **C**, Control diet; **HFHF**, high fat-high fructose diet; **HFHF +WPI**, high fat-high fructose diet + whey protein isolate; **C+WPI**, Control diet+ whey protein isolate. Results were determined by repeated measures analysis of variance (Repeated measures ANOVA). Values are given as mean with standard error of means (SEM). **a**, significant difference between C+WPI and HFHF (p<0.05); **b**, significant difference between C+WPI and HFHF+WPI (p<0.05); **c**, significant difference between C and HFHF (p<0.05).

Body weight changes of the rats over time are given in **Fig 1**. The changes in the body weights of the rats according to the diet–time interaction was statistically significant. (diet x time, p<0.001). The body weight of the HFHF group was significantly higher than the C+WPI group after the 6th week (p<0.05). In the final week, the body weight of the HFHF+WPI group was significantly higher than the C+WPI group, whereas the HFHF group was significantly higher than both the C and C+WPI groups (p<0.05).

### 3.2. Effects of whey protein isolate on biochemical profiles

**Fig 2:**
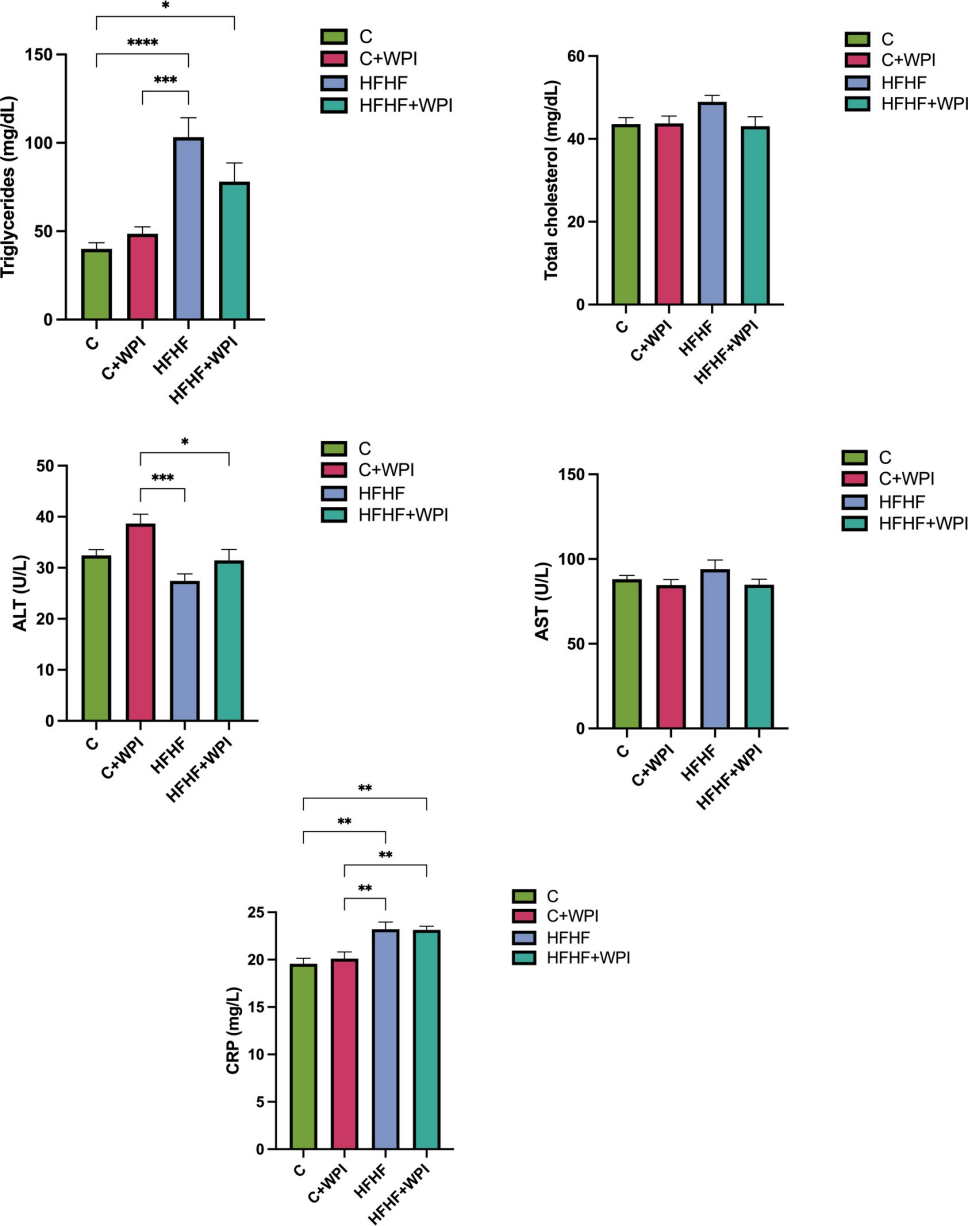
Whey protein supplementation did not alter the biochemical markers of male rats fed C, C+WPI, HFHF, or HFHF+WPI diets. Triglyceride levels of the HFHF group were significantly higher than C and C+WPI groups (p<0.001). The HFHF and HFHF+WPI groups had higher CRP levels than the C and C+WPI groups (p<0,05). **C**, Control diet; **HFHF**, high fat-high fructose diet; **HFHF +WPI**, high fat-high fructose diet + whey protein isolate; **C+WPI**, Control diet+ whey protein isolate. **ALT**, alanine transaminase; **AST**, aspartate transaminase; **CRP**, C-reactive protein. Results were determined by one-way analysis of variance (One-Way ANOVA) and expressed as mean and standard error of means (SEM). Tukey HSD test was used as post-hoc test in pairwise comparisons. * (p ≤ 0.05); ** (p ≤ 0.01); *** (p ≤ 0.001); **** (p ≤ 0.0001).

Mean values of serum triglyceride, cholesterol, ALT, AST, and CRP of rats are given in **Fig 2**. Triglyceride levels of the HFHF group were found to be significantly higher than C and C+WPI groups (p<0.001). The HFHF+WPI group had significantly higher triglyceride levels compared with the C group (p<0.05). No statistical difference was observed for cholesterol and AST levels between the groups. The C+WPI group had higher ALT values than the HFHF and HFHF +WPI groups (p<0.05). The HFHF and HFHF+WPI groups had higher CRP levels than the C and C+WPI groups (p<0.05).

**Fig 3:**
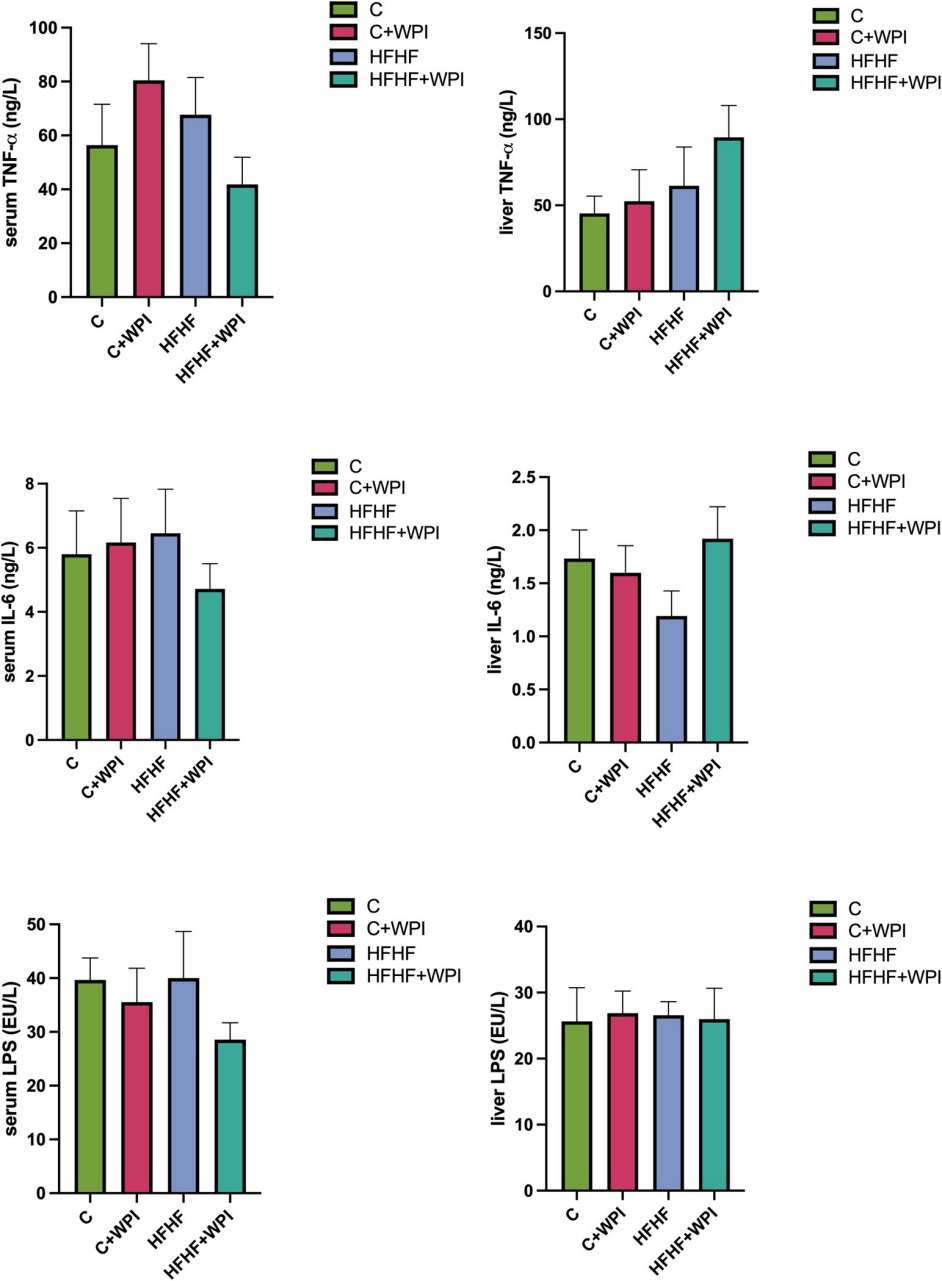
Whey protein supplementation did not alter the inflammatory markers of male rats fed C, C+WPI, HFHF, or HFHF+WPI diets. **C**, Control diet; **HFHF**, high fat-high fructose diet; **HFHF +WPI**, high fat-high fructose diet + whey protein isolate; **C+WPI**, Control diet+ whey protein isolate. Results were determined by one-way analysis of variance (One-Way ANOVA) and expressed as mean and standard error of means (SEM). Tukey HSD test was used as post-hoc test in pairwise comparisons. * (p ≤ 0.05); ** (p ≤ 0.01); *** (p ≤ 0.001); **** (p ≤ 0.0001).

**Fig 3** shows the levels of inflammatory markers and LPS in serum and liver. Serum LPS levels were found to be lower in the HFHF+WPI group compared with the HFHF group, but this difference was not significant (p>0.05). The group with the highest serum TNF-α value was found to be the C+WPI group, although there was no statistically significant difference between the groups (p>0.05). No significant difference was observed for the serum and liver IL-6 levels (p>0.05).

### 3.3. Effects of whey protein isolate histopathological findings

**Fig 4:**
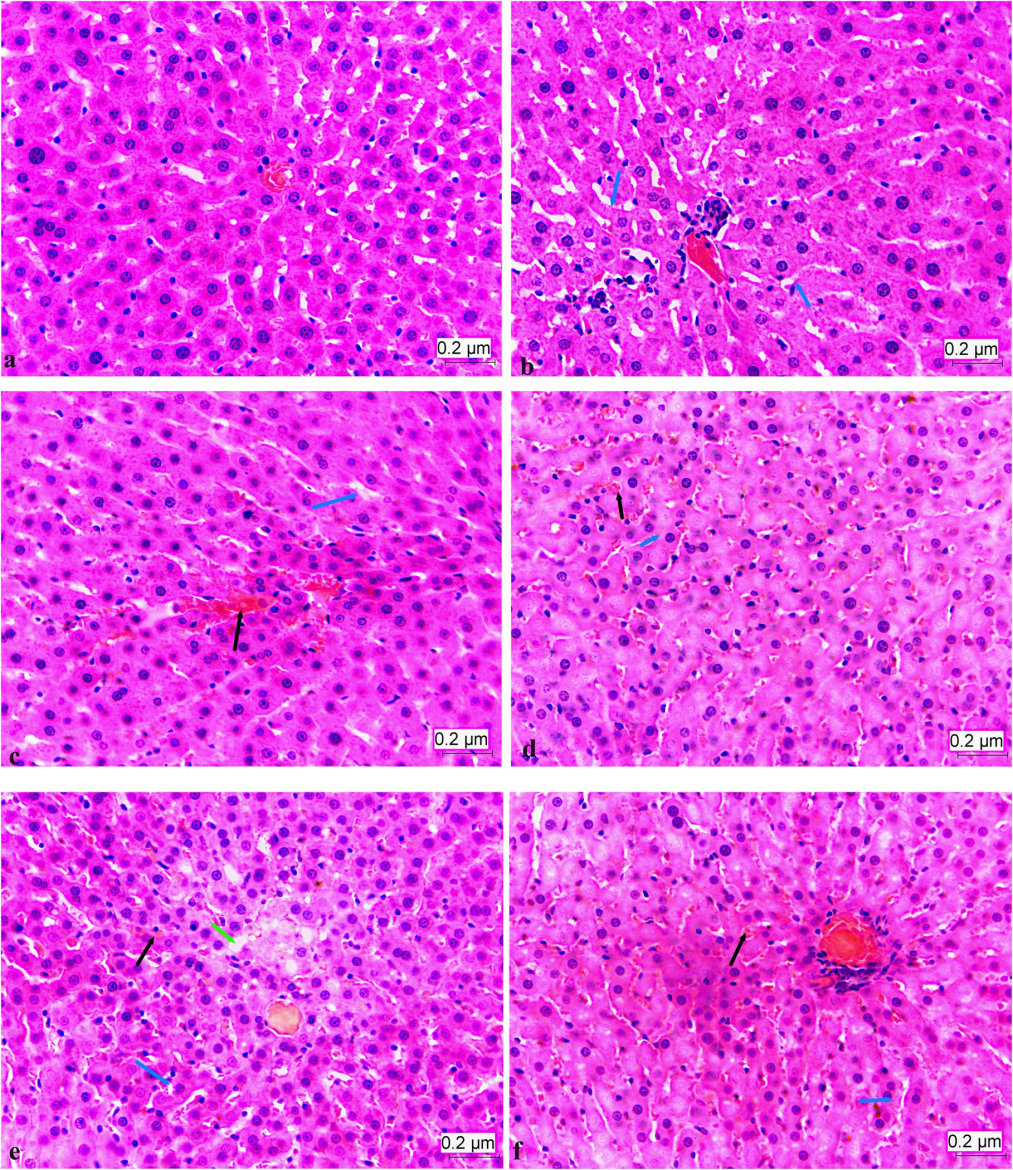
Whey protein supplementation lowered the liver damage of male rats fed on a HFHF diet. **a.** The normal structure of the liver in the cross-section of the C group (HE×40); **b.** Section of C+WPI group, sinusoidal expansion (blue arrows) in certain regions (HE×40); **c.** Section of HFHF+WPI group, hemopoietic areas (black arrow), occasional sinusoidal enlargement (blue arrow) (HE×40); **d**. Cross-sectional hemopoietic areas (black arrow), sinusoidal enlargement in places (blue arrow) belonging to group HFHF (HE×40); **e.** Section HFHF group, includes hemopoietic areas (black arrow), occasional sinusoidal enlargement (blue arrow), and necrotic areas (green arrow) (HE×40); **f.** Section of HFHF group, hemopoietic areas (black arrow), occasionally sinusoidal enlargement (blue arrow) (HE×40). **C**, Control diet; **HFHF**, high fat-high fructose diet; **HFHF +WPI**, high fat-high fructose diet + whey protein isolate; **C+WPI**, Control diet+ whey protein isolate.

**Fig 5:**
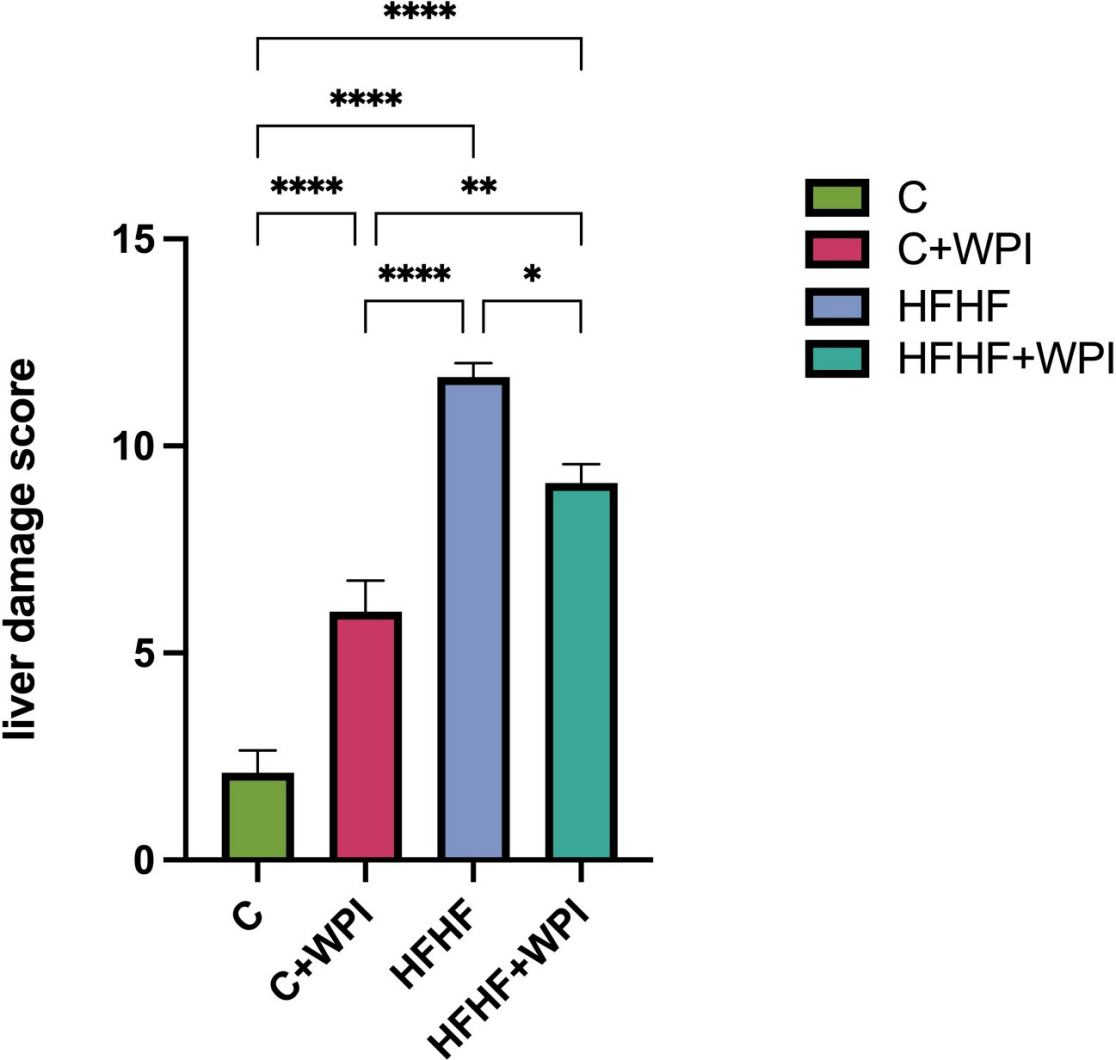
Whey protein supplementation lowered the total liver damage scores of male rats fed on a HFHF diet. **C**, Control diet; **HFHF**, high fat-high fructose diet; **HFHF +WPI**, high fat-high fructose diet + whey protein isolate; **C+WPI**, Control diet+ whey protein isolate. Results were determined by one-way analysis of variance (One-Way ANOVA) and values are given as mean with standard error of means (SEM). * (p ≤ 0.05); ** (p ≤ 0.01); *** (p ≤ 0.001); **** (p ≤ 0.0001).

**Fig 6:**
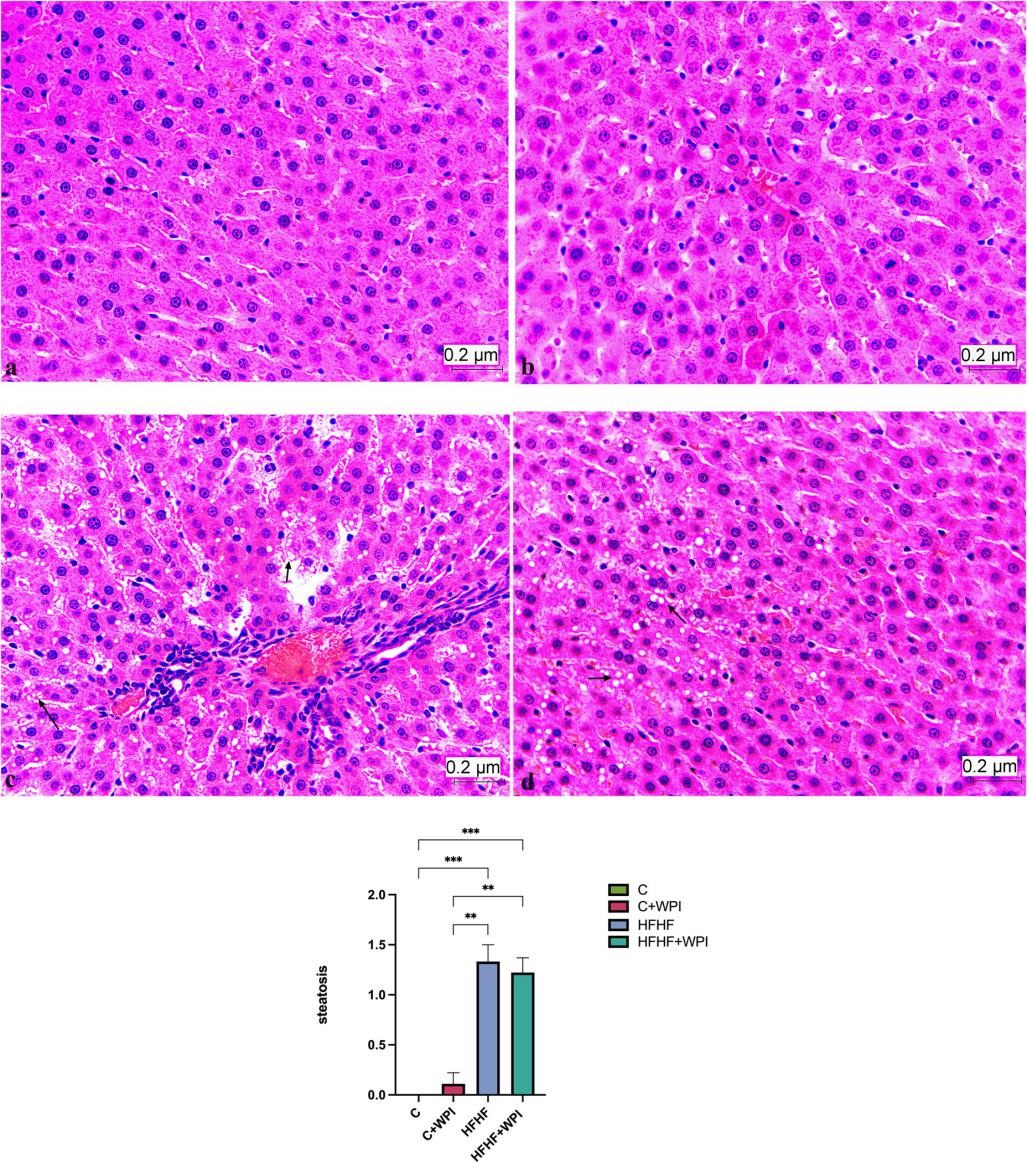
Whey protein supplementation did not alter the steatosis grading of male rats fed HFHF diet. The level of steatosis was higher in the HFHF and HFHF+WPI groups compared with C and C+WPI groups (p<0,05). **a. C**, Control diet (HE×40); **b**. **C+WPI**, Control diet+ whey protein isolate (HE×40); **c. HFHF**, high fat-high fructose diet (HE×40); **d**. **HFHF +WPI**, high fat-high fructose diet + whey protein isolate (HE×40); Results were determined by Kruskall-Wallis analysis. Values are given as mean with standard error of means (SEM). * (p ≤ 0.05); ** (p ≤ 0.01); *** (p ≤ 0.001); **** (p ≤ 0.0001).

H&E staining of liver sections are given in **Fig 4.** Hemopoietic areas at different densities, sinusoidal enlargement, hydropic degeneration, congestion, and necrotic areas were observed in the experimental groups (HFHF and HFHF+WPI). Mild inflammatory cell infiltrations were also observed in some sections of the HFHF group. For the total liver damage score, the HFHF group had the highest score followed by HFHF+WPI, C+WPI and C (p<0,05; **Fig 5**). For the steatosis grading, no statistical difference was observed between the HFHF and HFHF+WPI groups (p>0,05; **Fig 6**). The level of steatosis was higher in the HFHF and HFHF+WPI groups compared with C and C+WPI groups (p<0,05). No statistical difference was found between the C and C+WPI groups (p>0,05; **Fig 6**).

Intestine and colon sections of the groups are given in the **S1** and **S2 Figs**, respectively. Normal histological appearance was dominant in small intestine sections of the C group. Histological images of villi and crypts were normal. Similar to the C group, normal histological appearance was observed in small intestine sections in HFHF+WPI, HFHF, and C+WPI groups. Colon sections showed normal histological appearance for all of the groups.

### 3.4. Effects of whey protein isolate immunohistochemical findings

**Fig 7:**
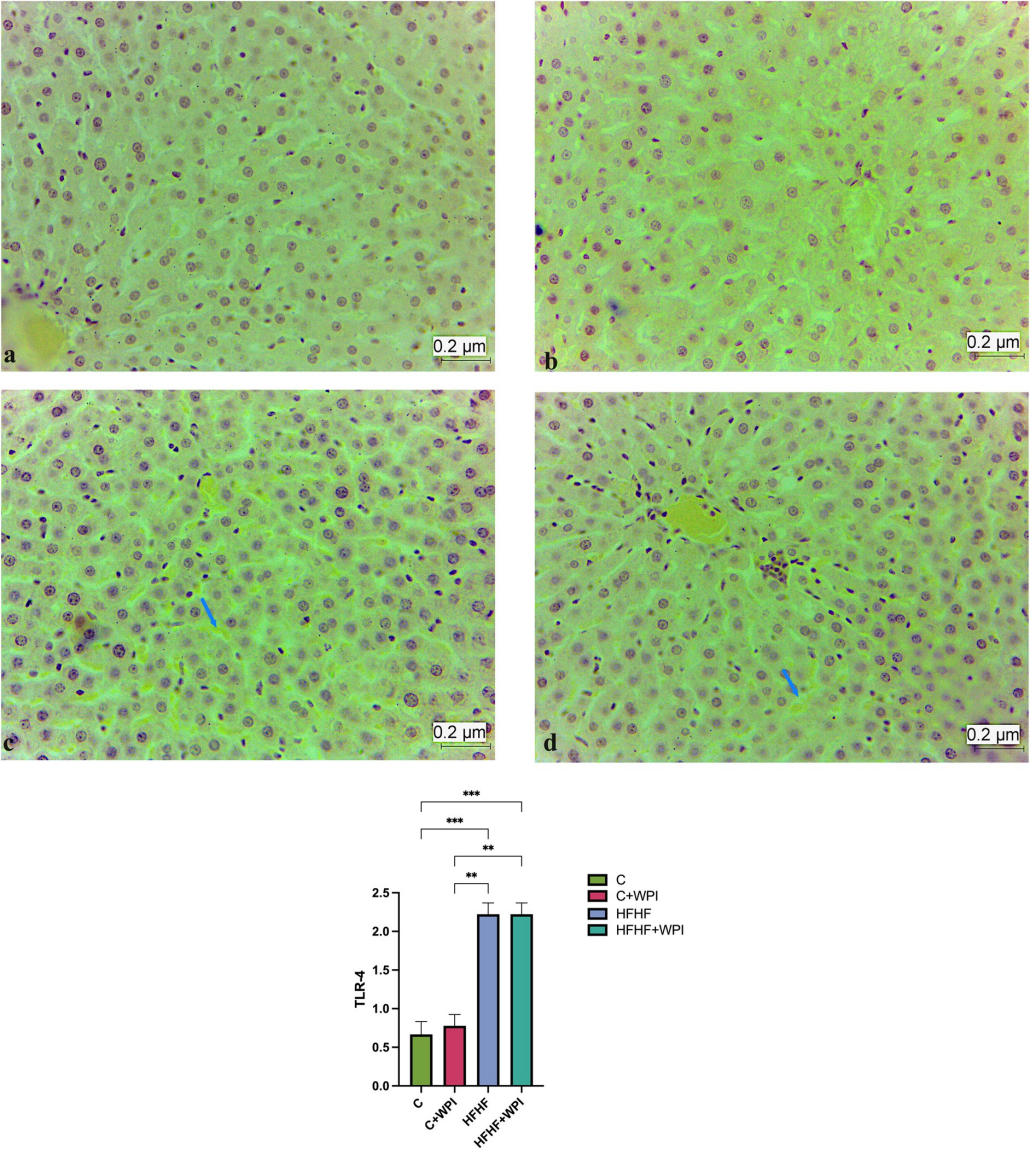
Whey protein supplementation did not alter the liver TLR-4 expression of male rats fed HFHF diet. TLR-4 expression in liver was higher in HFHF+WPI and HFHF groups compared with C+WPI and C groups (p<0,05). **C**, Control diet; **HFHF**, high fat-high fructose diet; **HFHF +WPI**, high fat-high fructose diet + whey protein isolate; **C+WPI**, Control diet+ whey protein isolate. **a.**TLR-4 immuno-negative cells in the liver tissue of the C group, 40; **b.** TLR-4 immuno-negative cells in liver tissue belonging to the C+WPI group, X 40; **c.** TLR-4 immuno-positive cells in liver tissue belonging to HFHF group, X 40; **d.** TLR-4 immuno-positive cells in liver tissue of HFHF+WPI group, X 40 Results were determined by Kruskall-Wallis analysis. Values are given as mean with standard error of means (SEM). * (p ≤ 0.05); ** (p ≤ 0.01); *** (p ≤ 0.001); **** (p ≤ 0.0001).

**Fig 8:**
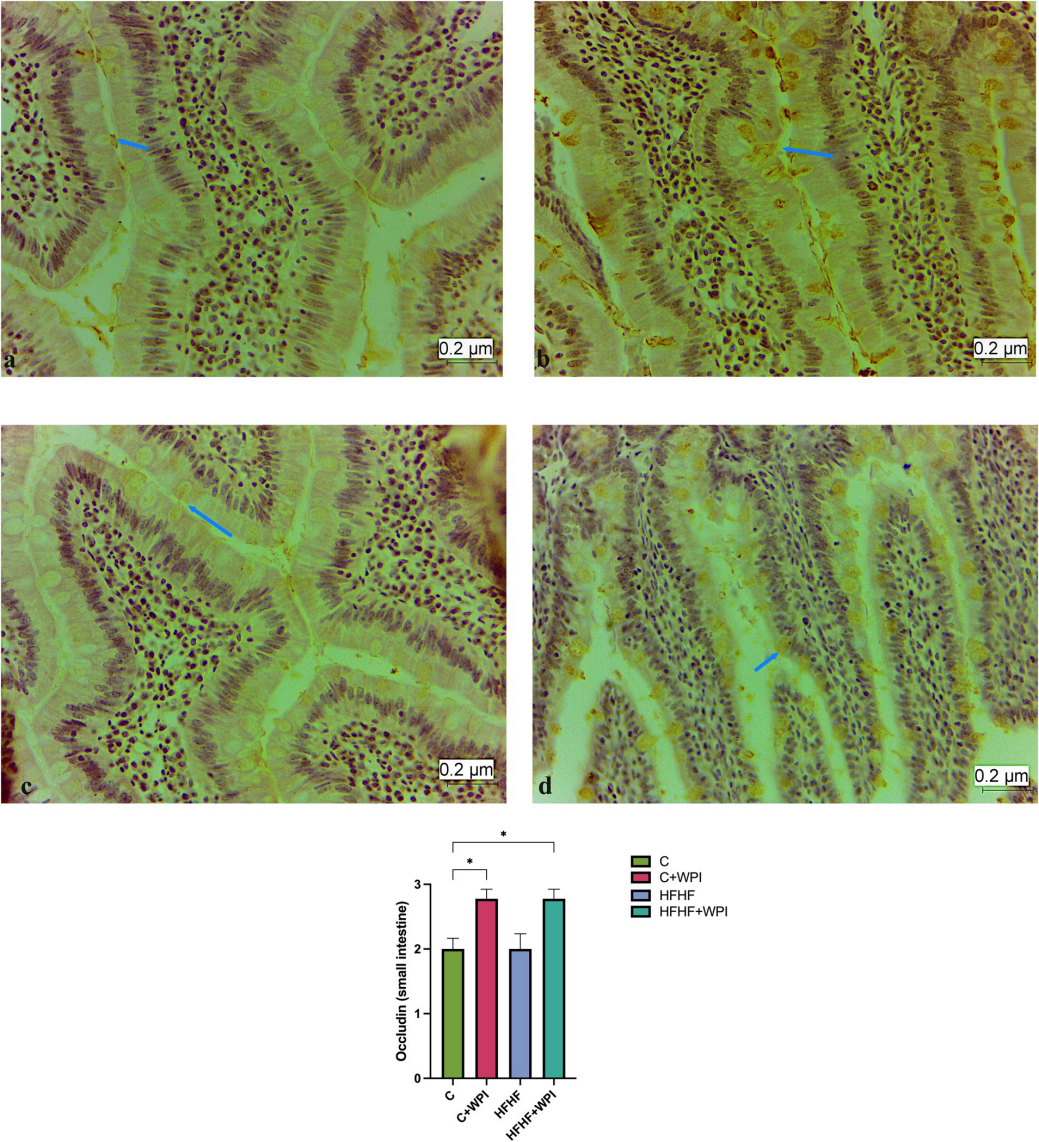
Whey protein supplementation increased the small intestine Occludin expression of male rats fed C and HFHF diet. **C**, Control diet; **HFHF**, high fat-high fructose diet; **HFHF +WPI**, high fat-high fructose diet + whey protein isolate; **C+WPI**, Control diet+ whey protein isolate. **a.** Occludin immuno-positive cells in the small intestine tissue of the C group, X 40; **b.** Occludin immuno-positive cells in the small intestine tissue of C+WPI group, X 40; **c.** Occludin immuno-positive cells in small intestinal tissue belonging to the HFHF group, X 40; **d.** Occludin immuno-positive cells in the small intestine tissue of the HFHF+WPI group, X 40. Results were determined by Kruskall-Wallis analysis. Values are given as mean with standard error of means (SEM). * (p ≤ 0.05); ** (p ≤ 0.01); *** (p ≤ 0.001); **** (p ≤ 0.0001).

**Fig 9:**
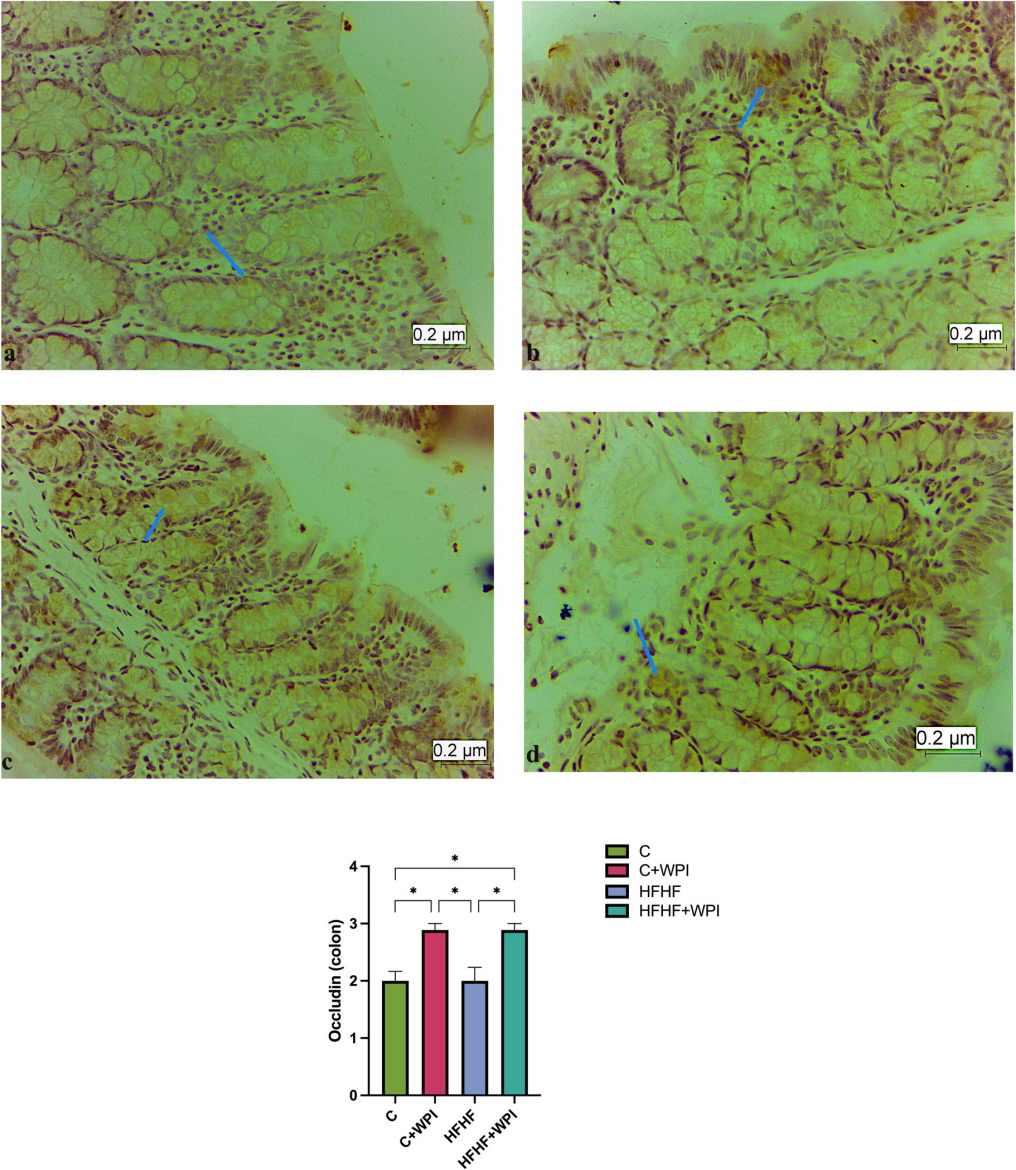
Whey protein supplementation increased the colon Occludin expression of male rats fed C and HFHF diet. **C**, Control diet; **HFHF**, high fat-high fructose diet; **HFHF +WPI**, high fat-high fructose diet + whey protein isolate; **C+WPI**, Control diet+ whey protein isolate. **a.** Occludin immune-positive cells in the colon tissue of the C group, X 40; **b.** Occludin immune-positive cells in colon tissue belonging to C+WPI group, X 40; **c.** Occludin immune-positive cells in colon tissue belonging to the HFHF group, X 40; **d.** Occludin immune-positive cells in colon tissue belonging to HFHF+WPI group, X 40. Results were determined by Kruskall-Wallis analysis. Values are given as mean with standard error of means (SEM). * (p ≤ 0.05); ** (p ≤ 0.01); *** (p ≤ 0.001); **** (p ≤ 0.0001).

Immunohistochemical findings of the liver are given in the **Fig 7**. TLR-4 expression in liver was higher in HFHF+WPI and HFHF groups compared with C+WPI and C groups (p<0,05). No significant difference was observed between the HFHF+WPI and HFHF groups in terms of TLR-4 expression (p>0,05). Immunohistochemical findings of the small intestine and colon are given in the **Figs 8** and **9**. Occludin expression in the small intestine was higher in the HFHF+WPI and C+WPI groups than in the C group. Small intestinal Occludin expression was found to be lower in the HFHF group than in the HFHF+WPI and C+WPI groups, but this difference was not significant (p>0,05; **Fig 8**). Occludin expression in the colon was found to be significantly higher in the HFHF+WPI and C+WPI groups than in HFHF and C groups. No significant difference was observed between HFHF and C groups (p>0,05; **Fig 9**).

## 4. Discussion

NAFLD is emerging as the most common chronic liver disease in the Western world [29]. Dietary management strategies for NAFLD focus on the metabolic risk factors and protecting the liver from oxidative stress [6,30,31]. Currently, different functional foods with bioactive nutrients are being sought for the management of NAFLD [6,32]. In the present study, we conducted an experimental model on rats with an HFHF diet to observe the effects of whey proteins on the liver, inflammatory markers, and intestinal permeability. In accordance with our hypothesis, whey protein supplementation reduced the liver damage associated with an HFHF diet. On the other hand, whey protein supplementation did not protect against liver steatosis caused by an HFHF diet and this hypothesis was rejected.

Our findings showed that WPI-treated rats in the HFHF+WPI diet group had a slightly lower body weight than the HFHF diet group, although this result was not statistically significant. On the other hand, the HFHF+WPI group had significantly lower abdominal circumference when compared with the HFHF group. Measurement of total body weight in rodents might not provide a relevant information about body composition, which is a key parameter in this type of research [33]. Alternatively, among various anthropometric measures, waist circumference was found to be the best predictor of intra-abdominal fat thickness, thus central obesity in humans [34]. A study conducted on rats suggests that abdominal circumference measurement might be a good tool for estimating visceral fat mass in rodents [33]. Previous studies have shown that whey protein supplementation may increase lean body mass and lower the adiposity in rodents and in humans [35–37]. For example, significant reduction in weight gain and adiposity was observed in mice fed with high-fat (HF) and WPI diet starting from the 5-weeks old. In the same study, when the procedure started with 10-weeks old mice, no significant difference was observed for the body weight of HF-WPI diet whereas lower weight of subcutaneous white adipose tissue (sWAT) was seen [38]. Therefore, these results might be explained by the changes in body composition, as whey protein supplementation could promote an increase in lean body mass and a decrease in adiposity [35,37,39]. In accordance with the study mentioned earlier above, we found lower abdominal circumference with WPI supplementation, although the body weight did not significantly differ between the groups [38]. Indeed a study on C57Bl/6J mice showed that WPI dose-dependently induced weight loss and this was mainly due to the changes in body fat content (e.g., decreased body fat percentage and decreased weight of fat pads) and not due to the changes in satiety or increased faecal fat excretion [22].

Inflammation and oxidative stress are associated with the development of obesity, dyslipidemia, and hepatic steatosis [40]. Although we observed higher expression of TLR-4 in the liver tissue of rats with an HFHF diet, we did not observe an increase in the pro-inflammatory cytokines in the serum samples. One possible explanation for this result might be that the animals used in our study were not under any significant chronic induction of the innate immune system [41]. Supporting our results, Feillet-Coudray et al. [40] observed only limited inflammation in HFHF-fed rats, whereas plasma levels of TNF-α and IL-6 remained unchanged. Therefore, despite long-term (for 20 weeks) HFHF diet feeding, inflammation status and oxidative stress remained very low for the HFHF diet in their study [40]. Similarly, in another study, HF diet for 12 weeks did not result with increased serum TNF-α and IL-6 levels [42]. Although low-grade inflammation might be associated with steatosis, overt inflammation is generally observed in more advanced stages of steatosis, and together with oxidative stress, is known to cause the progression of steatosis to steatohepatitis [40]. We observed higher CRP levels in HFHF and HFHF+WPI groups compared to C+WPI and C groups. Obesity and obesity-associated diseases are linked with elevated CRP levels [43]. Supporting our results, in another study, male Wistar rats fed an HF diet for 12 weeks showed higher CRP levels but no changes were detected in IL-6 and TNF-α levels [44]. There are studies showing an HF diet caused an increase in CRP levels but not in IL-6 and TNF-α levels [44,45] and one study [46] showed that there were no correlation between CRP levels and IL-6 levels as expected. We observed increased triglyceride levels with an HFHF diet, but ALT and AST levels were not affected. Similarly, an HF diet for 12 weeks showed no effects on ALT and AST levels in male Wistar rats [44].

Previous studies with experimental models of NAFLD showed that whey protein supplementation (in forms of whey protein concentrate or whey protein isolate) had protective effects against liver steatosis [31,47,48]. A study in HF diet-fed mice revealed that α-lactalbumin (a major component of whey protein) significantly upregulated the expression of lipid oxidation-related genes such as PPARα (peroxisome proliferator- activated receptor α, ACOX1 (Acetyl-CoA oxidase) and ATGL (adipose triglyceride lipase) in the liver while down-regulated the expression of lipid synthesis- related genes such as ACC1(Acetyl-CoA carboxylase) and SCD1 (Stearoyl-CoA desaturase). They concluded that α-lactalbumin exerts its action on hepatic steatosis and dyslipidemia by modulating the gene expressions related to lipid metabolism [48]. In contrast to these results from previous studies, we found that the whey protein supplementation had no effects on the liver steatosis. On the other hand, the HFHF+WPI group had a lower total liver damage score compared to the HFHF diet group. Therefore, although whey protein supplementation had not shown any effect on liver steatosis, total liver damage was reduced. A possible explanation for lower liver damage scores with whey protein supplementation could be due to the antioxidant effects of whey proteins. Whey proteins have a high content of sulfur-rich amino acids, particularly cysteine, a precursor to primary intracellular antioxidant, glutathione (GSH) [6]. Thus, the antioxidant effects of whey proteins are associated with their contribution to GSH synthesis [6,49]. The literature suggests that different cell lines treated with whey proteins exhibit increases in GSH levels [6,49–51]. It has been shown in Wistar rats that a diet supplemented with whey protein was protective against carbon tetrachloride (CCl4) hepatotoxicity by increasing serum levels of GSH [52]. Another study conducted on Wistar rats indicated that whey proteins have protective effects on the liver damage caused by ethanol [53]. Consistent with this finding, our results suggest that whey protein supplementation might be protective against liver damage, this time caused by the HFHF diet.

Over the last decade, the innate immune mechanisms in NAFLD have become the focus of research. It is suggested that changes in intestinal microbial balance and bacterial translocation might induce inflammatory responses in the liver through the signals derived from the intestines. Specifically, there is an increasing focus on pattern recognition receptors (PRRs) responsible for recognizing cell damage and pathogen invasion. Activation of PRRs prompts an inflammatory cascade that leads to cell injury in the liver and NAFLD progression. This inflammatory response involves the release of certain pro-inflammatory cytokines, including TNF-α and IL-6. One of the best characterized PRRs, Toll-like receptors (TLRs) are expressed in most liver cells. Under normal conditions, the liver plays a role in scavenging low levels of gut-derived LPS and other bacterial products (e.g., bacterial DNA, or other bacterial components) coming from portal circulation. As a result of impaired intestinal barrier function (e.g., increased intestinal permeability), the levels of LPS and other bacterial products increase in the portal circulation. This leads to overactivation of TLRs in the liver and induction of local inflammatory responses through the activation of NF-κB signalling. Increased activation of TLRs, specifically TLR-4 by binding of LPS, is suggested to be a key mechanism in liver inflammation and injury in NAFLD [54]. We investigated the effects of whey proteins on the gut–liver axis, mechanisms explained above. We found that an HFHF diet resulted with a higher liver TLR-4 expression, meanwhile serum levels of LPS remained unchanged. Similarly, previous studies also showed that an HF or HFHF diet induce higher expression of TLR-4 in rodents [47,55]. Consistent with our findings, a study revealed that C57Bl/6J mice fed an HF diet to induce obesity exhibited no differences in serum LPS levels compared to their lean counterparts [22]. On the other hand, there are also studies showing that an HF diet resulted with an increase in serum LPS levels [47,56]. In our study, whey protein supplementation showed no effects on TLR-4 expression, as no differences in expression were observed between the HFHF+WPI and HFHF diet groups. In the study conducted by Monteiro et al. [47], an HF diet caused an increase in TLR-4 expression and a combination of an HF diet with whey protein concentrate or hydrolysate for 9 weeks helped alleviate TLR-4 expression in mice. They concluded that whey proteins granted favorable conditions by preserving normal gut microbiota profile, help reduce the LPS levels in serum and alleviate liver fat infiltration [47]. In another murine study with an HF diet, α-lactalbumin (a major component of whey protein) helped alleviate TLR-4 expression [57]. These differences in results from our study might be explained by variability in the animals used in the experiments, the method of whey protein supplementation (e.g., introduction with the diet or supplementation through gavage), dose, or timing. Similar to our results, in an *in vitro* study,WPI showed no effect on the LPS-induced TLR-4 inflammatory pathway in HT29-MTX intestinal goblet cells [58]. Further studies are required to confirm these effects of whey proteins on TLR-4 expression with HF diets and specific mechanisms in which whey proteins exert their action.

Tight junctions (TJs) are cellular components that play a crucial role in maintaining barrier function in the intestine. They have complex molecular structures that create a barrier against external factors. Membrane components of TJs include; Occludin, Claudin, and Zonula Occludens (ZO) [59]. Some animal studies have suggested that an HF diet can impair intestinal permeability by inducing pro-inflammatory changes in the ileum and colon [60–62]. However, such increases in cytokine expression and histological findings with significant pro-inflammatory responses were not observed in all of the published studies [61,63]. Similarly, we did not observe any histopathological effects of the HFHF diet on the colon and intestinal tissues of the rats. In contrast, we found higher expression of Occludin in the HFHF+WPI and C+WPI groups compared with the HFHF and C groups. Therefore, whey protein supplementation resulted in a higher Occludin expression in both groups. In support of our results, in an *in vitro* study, commercial whey products (glycomacropeptide-GMP and WPI) promoted the upregulation of intestinal barrier function proteins such as Claudin and ZO-1. In this study, WPI particularly enhanced the up-regulation of Claudin-1 compared with GMP in Caco-2 cell lines [58]. One proposed explanation for this effect is that whey proteins contain high levels of transforming growth factor-β1 (TGF-β1), which has been reported to improve tight junction function [64]. TGF-β1 is known to show effects in critical biological processes, including epithelial cell growth and differentiation, immune regulation, and repairment of intestinal epithelium after injury [59]. In a previous *in vitro* study whey protein concentrate was shown to mediate its action on intestinal barrier function by inducing claudin-4 expression via TGF-β/Smad-4 signaling cascade. This study provides interesting insights on the molecular mechanism by which whey protein exert its effect on intestinal epithelial integrity [65]. Besides Smad-dependent pathways, the action of TGF-β1 is also associated with mithogen-activated protein kinase (ERK) and c-jun N-terminal kinase (JNK) pathways [59]. A study [59] explored the effects of WPC (whey protein concentrate) on LPS-induced intestinal injury in piglets. Together with the improved morphology and barrier function, WPC prevented the decrease in claudin-1, Occludin and ZO-1 expression. The authors also proposed that the WPC supplementation was responsible for increased expression of TGF-β1 as TGF-β1 in the diet can be absorbed by the intestine *in vivo*. They also confirmed the previous research as they observed activated Smad signaling pathways. In addition, they concluded that the protective effects of WPC on intestinal integrity after exposure to LPS challenge are also related to activation of ERK 1/2 and inhibition of JNK and p38 MAPK signaling pathways [59]. Higher Occludin expression in the groups with WPI supplementation in our study supports the previous findings on the possible promoting effects of whey proteins on intestinal barrier integrity [59,64–67]. Further research is necessary to confirm the mechanisms by which whey proteins improve intestinal integrity. Well-designed clinical trials using whey proteins could be a potential interest for diseases characterized by intestinal barrier dysfunction [64].

Generally, whey proteins are considered a safe supplement as not many side effects have been reported in previous studies [6,30,68–71]. Although the intake of active peptides coming from a normal diet is generally considered safe, excessive administration of them can have adverse consequences. For this reason, peptides that are not from basic foods should be evaluated in terms of toxicology [6,72]. Some concerns associated with an overdose of whey proteins include possible adverse effects on kidney and liver function [73,74]. Although these side effects have been reported in some studies [75,76], the available data are still limited to draw firm conclusions [77]. Interestingly, in our study we found higher liver damage scores with whey protein isolate administration (200 mg/kg) to a control diet for 8 weeks. Another study with healthy rats supplemented with whey protein concentrate (WPC-80) at a dose of 0.3 g/kg for 21 days showed hepatocellular damage with a loss of nuclei. More extensive damage was observed in rats fed with a higher dose (0.5g/kg) of WPC-80. However, no signs of inflammation and fibrosis were observed by histological examination. The authors also found increased glutathione biosynthesis, but they concluded that this increase was not effectively protective against the reactive oxygen species (ROS) production under the influence of increased protein supply [78]. Further studies are required to explain the observed changes and underlying mechanisms for these results from the previous study. It is particularly important to properly adjust the dose and duration of the whey protein supplementation [78]. Therefore, more data are needed to investigate the use of different doses in both short and long-term studies regarding the aforementioned safety issues [6,74].

Finally, we would like to acknowledge some limitations in our study. Firstly, intestinal microbiota analysis was not performed. Thus, we could not assess the changes between intestinal microbiota composition among the groups and effects of whey protein supplementation. Another limitation is that we did not assess the antioxidant enzyme activity in liver tissues and oxidative stress markers. We believe that assessment of these parameters would increase our understanding of the antioxidant effects of whey proteins. Finally, TLR-4 expression in liver tissues and Occludin expression in intestinal and colonic tissues was evaluated by immunohistochemical analysis alone. In addition to immunohistochemical analysis, it is important to demonstrate the expression of TLR-4 and Occludin by immunoblotting, as it is a more sensitive method.

### 4.1 Conclusion

Our study provides some interesting insights into the effects of whey proteins on intestinal barrier function and NAFLD-related liver damage. The findings of this study suggest that simultaneous whey protein supplementation with an HFHF diet have positively affects the liver damage. Further studies are required to confirm and validate the effects of whey proteins on liver damage. The duration of administration and the optimal dose should be tested further to determine the optimal conditions for achieving beneficial results. Interestingly, we also observed an increased total liver damage score with whey protein isolate supplementation to a control diet. Therefore, further studies should address the possible side effects and safety of whey proteins from long-term administration. Whey protein isolate supplementation showed no effects on the steatosis induced with an HFHF diet. Another striking finding was that whey protein supplementation resulted in an increase in Occludin expression, which indicates an increase in the intestinal barrier function. Further clarification is required for the mechanism by which whey proteins potentially upregulate Occludin expression. The final goal is to translate these approaches to human nutrition with clinical studies and develop new strategies based on functional foods.

## Supporting information

S1 Fig Small intestine sections of groups. **a.** Normal structure of the small intestine in the section of the C group (HE×40); **b**. Normal structure of the small intestine in the section of the C+WPI group (HE×40); **c.** Normal structure of the small intestine in the section of the HFHF+WPI group (HE×40); **d.** Normal structure of the small intestine in the section of the HFHF group (HE×40).
(DOCX)

S2 Fig Colon sections of groups. **a**. Normal structure of the colon in the section from the C group (HE×40); **b.** Normal structure of the column in the section from the C+WPI group (HE×40); **c.** Normal structure of the column in the section from the HFHF+WPI group (HE×40); **d.** Normal structure of the column in the section from the HFHF group (HE×40).
(DOCX)

S1 Table Composition of the whey protein isolate. (DOCX)

S2 Table Dataset of the levels of some biochemical parameters of the groups. **HFHF +WPI**, high fat-high fructose diet + whey protein isolate; **C+WPI**, Control diet+ whey protein isolate; **HFHF**, high fat-high fructose diet; **C**, Control diet; **ALT**, alanine transaminase; **AST**, aspartate transaminase; **CRP**, C-reactive protein. Results were determined by one-way analysis of variance (One-Way ANOVA) and expressed as mean and standard error of means. Tukey HSD test was used as post-hoc test in pairwise comparisons. Different letters indicate statistical significance.
(DOCX)

S3 Table Dataset of the levels of inflammatory parameters and endotoxin of the groups. **HFHF +WPI**, high fat-high fructose diet + whey protein isolate; **C+WPI**, Control diet+ whey protein isolate; **HFHF**, high fat-high fructose diet; **C**, Control diet; **TNF-α**, tumor necroes factor-α; **IL-6**, interleukin-6; **LPS**, lipopolysaccharide. Results were determined by one-way analysis of variance (One-Way ANOVA) and expressed as mean and standard error of means. Tukey HSD test was used as post-hoc test in pairwise comparisons. Different letters indicate statistical significance.
(DOCX)

S4 Table Dataset of the total liver damage scores of the groups. **HFHF +WPI**, high fat-high fructose diet + whey protein isolate; **C+WPI**, Control diet+ whey protein isolate; **HFHF**, high fat-high fructose diet; **C**, Control diet. Results were determined by one-way analysis of variance (One-Way ANOVA) and expressed as mean and standard error of means. Tukey HSD test was used as post-hoc test in pairwise comparisons. Different letters indicate statistical significance.
(DOCX)

S5 Table Dataset of the steatosis levels of the groups. **HFHF +WPI**, high fat-high fructose diet + whey protein isolate; **C+WPI**, Control diet+ whey protein isolate; **HFHF**, high fat-high fructose diet; **C**, Control diet. Results were determined by Kruskall- Wallis analysis and expressed as mean, standard error of means, minimum, maximum and quarter. Different letters indicate statistical significance.
(DOCX)

S6 Table Dataset of the TLR-4 and Occludin expression levels of the groups. **HFHF +WPI**, high fat-high fructose diet + whey protein isolate; **C+WPI**, Control diet+ whey protein isolate; **HFHF**, high fat-high fructose diet; **C**, Control diet. Results were determined by Kruskall- Wallis analysis and expressed as mean, standard error of means, minimum, maximum and quarter. Different letters indicate statistical significance.
(DOCX)

## Abbreviations

AC: abdominal circumference
ALT: alanine transaminase
AST: aspartate transaminase
BMI: body mass index
CCl4: carbon tetrachloride
C: control diet
C+WPI: Control diet+ whey protein isolate
CRP: C-reactive protein
ELISA: Enzyme-linked Immunosorbent Assay
GMP: glycomacropeptide
GSH: glutathione
HF: high-fat; HFHF, high fat-high fructose diet
WPI: whey protein isolate
HFHF+WPI: high fat-high fructose diet + whey protein isolate
IL-1β: interleukin 1β
IL-6: interleukin-6
LPS: lipopolysaccharide
NAFLD: Non-alcoholic fatty liver disease
NASH: non-alcoholic steatohepatitis
PRRs: pattern recognition receptors
ROS: Reactive oxygen species
sWAT: subcutaneous white adipose tissue
TJs: Tight junctions
TGFβ: transforming growth factor-β1
TLRs: toll-like receptors
TLR-4: toll-like receptor-4
TNF-α: tumor necroes factor-α
WPC: whey protein concentrate
ZO: zonula occludens.
