# Supplementary material for: Whey protein supplementation reduced the liver damage scores of rats fed with a high fat-high fructose diet
Source: PLoS One. 2024 Apr 4;19(4):e0301012. doi: 10.1371/journal.pone.0301012 (PMC10994406; doi:10.1371/journal.pone.0301012)
Supplement: S1 Fig — a. Normal structure of the small intestine in the section of the C group (HE×40); b. Normal structure of the small intestine in the section of the C+WPI group (HE×40); c. Normal structure of the small intestine in the section of the HFHF+WPI group (HE×40); d. Normal structure of the small intestine in the section of the HFHF group (HE×40). (DOCX) [file pone.0301012.s001.docx]

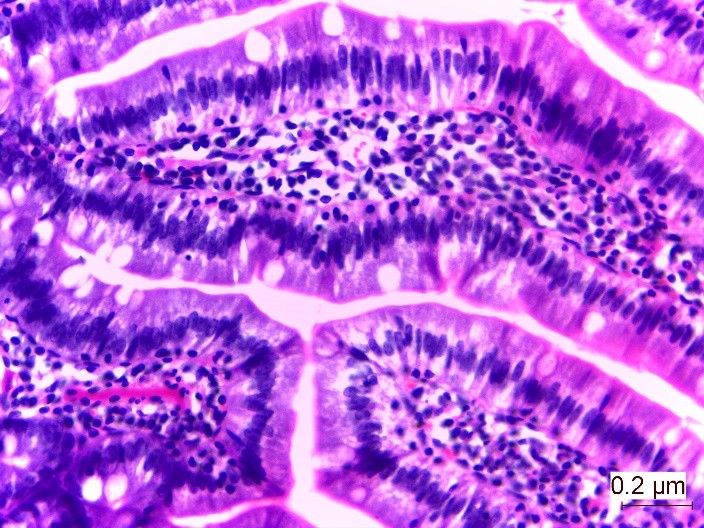


a


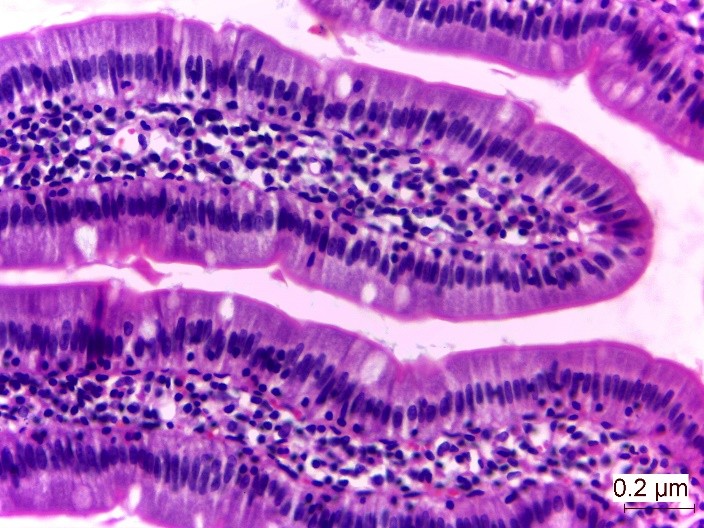


b


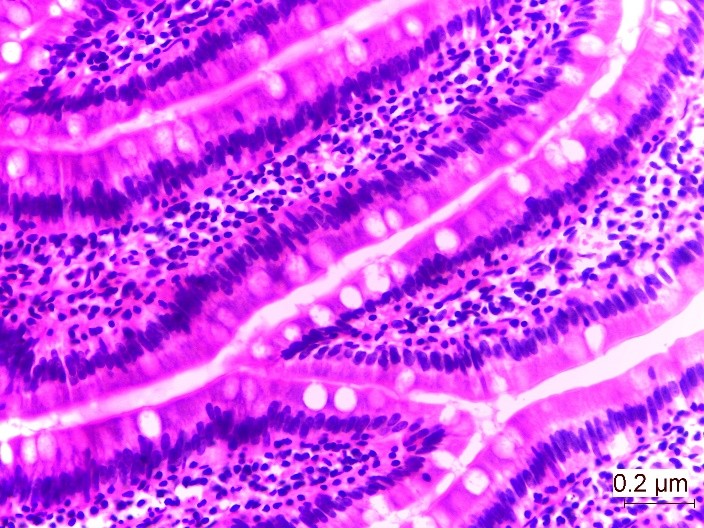


c


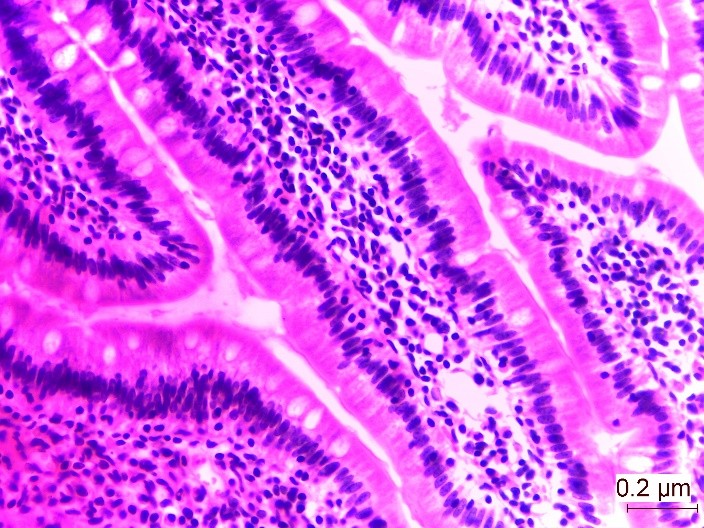


d

**S1 Fig.** Small intestine sections of groups**. a.** Normal structure of the small intestine in the section of the C group (HE×40); **b**. Normal structure of the small intestine in the section of the C+WPI group (HE×40); **c.** Normal structure of the small intestine in the section of the HFHF+WPI group (HE×40); **d.** Normal structure of the small intestine in the section of the HFHF group (HE×40).
