# Supplementary material for: Whey protein supplementation reduced the liver damage scores of rats fed with a high fat-high fructose diet
Source: PLoS One. 2024 Apr 4;19(4):e0301012. doi: 10.1371/journal.pone.0301012 (PMC10994406; doi:10.1371/journal.pone.0301012)
Supplement: S1 Table — (DOCX) [file pone.0301012.s003.docx]

**S1 Table. Composition of the whey protein isolate**

**(100 gr)**

Energy 377 kcal

Fat 0,7 g

Saturated Fat 0,42 g

Carbohydrate 4 g

Sugar 3,1 g

Fiber 0 g

Protein 89 g

Salt 0,89 g

| **Amino Acid Content** | **(100 gr)** |
| --- | --- |
| Alanine | 4,0 g |
| Arginine | 2,3 g |
| Aspartic acid | 11,1 g |
| Glutamic acid | 15,7 g |
| Cysteine | 2,8 g |
| Glycine | 1,7 g |
| Histidine | 1,7 g |
| Isoleucine | 4,5 g |
| Leucine | 9,0 g |
| Valine | 4,5 g |
| Lysine | 9,4 g |
| Methionine | 2,0 g |
| Phenylalanine | 3,3 g |
| Tryptophan | 2,2 g |
| Tyrosine | 3,4 g |
| Threonine | 4,6 g |
| Serine | 4,0 g |
| **Bioactive Protein Components**  α-lactalbumin | 14,1 g |
| β-lactoglobulin | 54,1 g |
| IgG | 1,9 g |
| lactoferrin | 140 mg |
