# Supplementary material for: Whey protein supplementation reduced the liver damage scores of rats fed with a high fat-high fructose diet
Source: PLoS One. 2024 Apr 4;19(4):e0301012. doi: 10.1371/journal.pone.0301012 (PMC10994406; doi:10.1371/journal.pone.0301012)
Supplement: S2 Table — HFHF +WPI, high fat-high fructose diet + whey protein isolate; C+WPI, Control diet+ whey protein isolate; HFHF, high fat-high fructose diet; C, Control diet; ALT, alanine transaminase; AST, aspartate transaminase; CRP, C-reactive protein. Results were determined by one-way analysis of variance (One-Way ANOVA) and expressed as mean and standard error of means. Tukey HSD test was used as post-hoc test in pairwise comparisons. Different letters indicate statistical significance. (DOCX) [file pone.0301012.s004.docx]

|  | **HFHF+WPI** | **C+WPI** | **HFHF** | **C** | **p** |
| --- | --- | --- | --- | --- | --- |
| Triglyceride (mg/dL) | 78,05 ± 10,60^ac^ | 48,57 ± 3,91^ab^ | 103,20 ± 10,97^c^ | 40,02 ± 3,43^b^ | **,000** |
| Cholesterol (mg/dL) | 43,05 ± 2,31 | 43,72 ± 1,80 | 48,94 ± 1,57 | 43,56 ± 1,56 | ,100 |
| AST  (U/L) | 84,82 ± 3,29 | 84,65 ± 3,20 | 93,98 ± 5,48 | 88,07 ± 2,20 | ,274 |
| ALT  (U/L) | 31,43 ± 2,15^a^ | 38,67 ± 1,83^b^ | 27,41 ± 1,39^a^ | 32,42 ± 1,12^ab^ | ,**001** |
| CRP  (mg/L) | 23,14 ± 0,37^a^ | 20,10 ± 0,70^b^ | 23,20 ± 0,76^a^ | 19,56 ± 0,58^b^ | ,**000** |

**S2 Table.** Dataset of the levels of some biochemical parameters between the groups.
