# Supplementary material for: Whey protein supplementation reduced the liver damage scores of rats fed with a high fat-high fructose diet
Source: PLoS One. 2024 Apr 4;19(4):e0301012. doi: 10.1371/journal.pone.0301012 (PMC10994406; doi:10.1371/journal.pone.0301012)
Supplement: S3 Table — HFHF +WPI, high fat-high fructose diet + whey protein isolate; C+WPI, Control diet+ whey protein isolate; HFHF, high fat-high fructose diet; C, Control diet; TNF-α, tumor necroes factor-α; IL-6, interleukin-6; LPS, lipopolysaccharide. Results were determined by one-way analysis of variance (One-Way ANOVA) and expressed as mean and standard error of means. Tukey HSD test was used as post-hoc test in pairwise comparisons. Different letters indicate statistical significance. (DOCX) [file pone.0301012.s005.docx]

|  | **HFHF+WPI** | **C+WPI** | **HFHF** | **C** | **p** |
| --- | --- | --- | --- | --- | --- |
| Serum TNF-α (ng/L) | 41,80 ± 10,11 | 80,46 ±13,59 | 67,67 ± 13,83 | 56,38 ± 15,15 | 0,226 |
| Liver TNF-α (ng/L) | 89,44 ± 18,55 | 52,33 ± 18,29 | 61,33 ± 22,53 | 45,26 ± 10,07 | 0,338 |
| Serum IL-6 (ng/L) | 4,71 ± 0,78 | 6,16 ± 1,37 | 6,45 ± 1,37 | 5,79 ± 1,35 | 0,775 |
| Liver IL-6 (ng/L) | 1,91 ± 0,30 | 1,59 ± 0,25 | 1,18 ± 0,23 | 1,73 ± 0,26 | 0,275 |
| Serum LPS (EU/L) | 28,54 ± 3,13 | 35,53 ± 6,30 | 39,99 ± 8,66 | 39,65 ± 4,09 | 0,502 |
| Liver LPS (EU/L) | 25,97 ± 4,66 | 26,87 ± 3,36 | 26,56 ± 2,05 | 25,64 ± 5,10 | 0,996 |

**S3 Table.** Dataset of the levels of inflammatory parameters and endotoxin between the groups
