# Supplementary material for: Whey protein supplementation reduced the liver damage scores of rats fed with a high fat-high fructose diet
Source: PLoS One. 2024 Apr 4;19(4):e0301012. doi: 10.1371/journal.pone.0301012 (PMC10994406; doi:10.1371/journal.pone.0301012)
Supplement: S4 Table — HFHF +WPI, high fat-high fructose diet + whey protein isolate; C+WPI, Control diet+ whey protein isolate; HFHF, high fat-high fructose diet; C, Control diet. Results were determined by one-way analysis of variance (One-Way ANOVA) and expressed as mean and standard error of means. Tukey HSD test was used as post-hoc test in pairwise comparisons. Different letters indicate statistical significance. (DOCX) [file pone.0301012.s006.docx]

**S4 Table.** Dataset of the total liver damage scores of the groups

|  | **HFHF+WPI** | **C+WPI** | **HFHF** | **C** | **p** |
| --- | --- | --- | --- | --- | --- |
| Total liver damage score | 9,11 ± 0,45^a^ | 6,00 ± 0,74^b^ | 11,67 ± 0,33^c^ | 2,11 ± 0,53^d^ | ,000 |
