# Supplementary material for: Whey protein supplementation reduced the liver damage scores of rats fed with a high fat-high fructose diet
Source: PLoS One. 2024 Apr 4;19(4):e0301012. doi: 10.1371/journal.pone.0301012 (PMC10994406; doi:10.1371/journal.pone.0301012)
Supplement: S5 Table — HFHF +WPI, high fat-high fructose diet + whey protein isolate; C+WPI, Control diet+ whey protein isolate; HFHF, high fat-high fructose diet; C, Control diet. Results were determined by Kruskall- Wallis analysis and expressed as mean, standard error of means, minimum, maximum and quarter. Different letters indicate statistical significance. (DOCX) [file pone.0301012.s007.docx]

**S5 Table.** Dataset of the steatosis levels of the groups

|  |  | **Mean ± SEM** | **Median** | **Minimum** | **Maximum** | **Quarter (25-75)** | **p** |
| --- | --- | --- | --- | --- | --- | --- | --- |
| Steatosis | HFHF+WPI | 1,22 ± 0,14^a^ | 1,00 | 1,00 | 2,00 | 1,00-1,50 | **0,000** |
|  | C+WPI | 0,11 ± 0,11^b^ | 0,00 | 0,00 | 1,00 | 0,00-0,00 |  |
|  | HFHF | 1,33 ± 0,16^a^ | 1,00 | 1,00 | 2,00 | 1,00-2,00 |  |
|  | C | 0,00 ± 0,00^b^ | 0,00 | 0,00 | 0,00 | 0,00-0,00 |  |
