# Supplementary material for: Whey protein supplementation reduced the liver damage scores of rats fed with a high fat-high fructose diet
Source: PLoS One. 2024 Apr 4;19(4):e0301012. doi: 10.1371/journal.pone.0301012 (PMC10994406; doi:10.1371/journal.pone.0301012)
Supplement: S6 Table — HFHF +WPI, high fat-high fructose diet + whey protein isolate; C+WPI, Control diet+ whey protein isolate; HFHF, high fat-high fructose diet; C, Control diet. Results were determined by Kruskall- Wallis analysis and expressed as mean, standard error of means, minimum, maximum and quarter. Different letters indicate statistical significance. (DOCX) [file pone.0301012.s008.docx]

**S6 Table.** Dataset of the TLR-4 and Occludin expression levels of the groups

|  |  | **Mean ± SEM** | **Median** | **Minimum** | **Maximum** | **Quarter (25-75)** | **p** |
| --- | --- | --- | --- | --- | --- | --- | --- |
| TLR-4 (liver) | HFHF+WPI | 2,22 ± 0,14^a^ | 2,00 | 2,00 | 3,00 | 2,00-2,50 | **0,000** |
|  | C+WPI | 0,78 ± 0,14^b^ | 1,00 | 0,00 | 1,00 | 0,50-1,00 |  |
|  | HFHF | 2,22 ± 0,14^a^ | 2,00 | 2,00 | 3,00 | 2,00-2,50 |  |
|  | C | 0,67 ± 0,17^b^ | 1,00 | 0,00 | 1,00 | 0,00-1,00 |  |
| Occludin (small intestine) | HFHF+WPI | 2,78 ± 0,14^a^ | 3,00 | 2,00 | 3,00 | 2,50-3,00 | **0,003** |
|  | C+WPI | 2,78 ± 0,14^a^ | 3,00 | 2,00 | 3,00 | 2,50-3,00 |  |
|  | HFHF | 2,00 ±0,23^ab^ | 2,00 | 1,00 | 3,00 | 1,50-2,50 |  |
|  | C | 2,00 ±0,16^b^ | 2,00 | 1,00 | 3,00 | 2,00-2,00 |  |
| Occludin (colon) | HFHF+WPI | 2,89 ± 0,11^a^ | 3,00 | 2,00 | 3,00 | 2,50-3,00 | **0,000** |
|  | C+WPI | 2,89 ± 0,11^a^ | 3,00 | 2,00 | 3,00 | 3,00-3,00 |  |
|  | HFHF | 2,00 ± 0,23^b^ | 2,00 | 1,00 | 3,00 | 1,50-2,50 |  |
|  | C | 2,00 ±0,16^b^ | 2,00 | 1,00 | 3,00 | 0,00-1,00 |  |
